# Repository scale classification and decomposition of tandem mass spectral data

**DOI:** 10.1038/s41598-021-87796-6

**Published:** 2021-04-15

**Authors:** Mihir Mongia, Hosein Mohimani

**Affiliations:** Computational Biology Department in the School of Computer Science, Carnegie Mellon University, Pittsburgh, USA

**Keywords:** Data mining, Machine learning

## Abstract

Various studies have shown associations between molecular features and phenotypes of biological samples. These studies, however, focus on a single phenotype per study and are not applicable to repository scale metabolomics data. Here we report MetSummarizer, a method for predicting (i) the biological phenotypes of environmental and host-oriented samples, and (ii) the raw ingredient composition of complex mixtures. We show that the aggregation of various metabolomic datasets can improve the accuracy of predictions. Since these datasets have been collected using different standards at various laboratories, in order to get unbiased results it is crucial to detect and discard standard-specific features during the classification step. We further report high accuracy in prediction of the raw ingredient composition of complex foods from the Global Foodomics Project.

## Introduction

Small molecules play a crucial role in the mechanisms behind diseases^1^. Untargeted tandem mass spectrometry provides an inexpensive way for capturing the fingerprints of known and novel small molecules and thus allows for the development of comprehensive mass spectral libraries such as Global Natural Product Social (GNPS) molecular networking infrastructure library^2^. GNPS has facilitated identification of all known small molecules from LC-MS/MS of complex samples through spectral library search. Moreover, GNPS has provided a repository for storing annotated metabolomics data and since its launch in 2016, over a million samples from five hundred laboratories have been uploaded to this repository. Currently, the majority of datasets from MetaboLights^3^ and NIH Metabolomics Workbench^4^ are imported to GNPS. In an effort to make metabolomics data as reusable as genomics data, Reanalysis of Data User Interface (ReDU) keeps record of the metadata for a subset of 34,087 samples from publicly available datasets on GNPS^5^. Availability of these large scale annotated datasets paves the path toward a better understanding of the relationships between molecular features and biological phenotypes.

In the past, various studies have shown the associations between small molecules and phenotypes. However, these studies focus on a single phenotype and thus are not applicable to repository scale data. In this paper we apply various machine learning methods on metabolomics data for prediction of phenotypes annotated as part of the ReDU project^5^. These phenotypes include age, biological sex, life-stage, and also diseases such as sleep deprivation, obesity, inflammatory bowel disease, and hypertension. We show that by aggregating data from various labs, machine learning can achieve far more accurate predictions than what is possible from a single dataset and this in turn enables accurate predictions of hundreds of other biological phenotypes. A challenging problem is that datasets originating from different labs have different protocols and varying internal standards. We recruit an interpretable machine learning technique where the bias can be detected and removed. This technique is further capable of revealing the molecular mechanism of disease.

Another challenging task in summarizing metabolomics samples is predicting the raw ingredients of complex mixtures. Inferring the compositions of mixtures is an important problem in domains such as water and air quality control^6,7^, microbiome analysis^8^, and food ingredient analysis. In the case of microbial community analysis, given metabolomics profile of a microbial community along with a reference database of the metabolomic profiles of isolated microbial strains, the goal is to predict the abundance of each of the strains, along with their contribution to each molecular feature. In the case of food ingredient analysis, given a complex dish, the goal is to predict its ingredients along with their abundances. These tasks are challenging because the metabolomic profile of various food ingredients (various isolated microbial strains) usually share many molecules. Therefore, it is not clear from which raw ingredient (which isolated microbial strain) the molecules in complex dishes (microbial communities) originate. Currently, computational techniques for predicting the ingredients of complex mixtures and their abundances based on mass spectral data are not available. We frame inferring ingredients of a complex mixture as an optimization problem, where the objective is to find a small number of ingredients whose combination is most similar to the query. Benchmarking our method on data from the Global FoodOmics Project^3–10^ shows a remarkable consistency between the ingredients reported by MetSummarizer and the known ingredients.

## Results

### Outline of MetSummarizer

**Figure 1:**
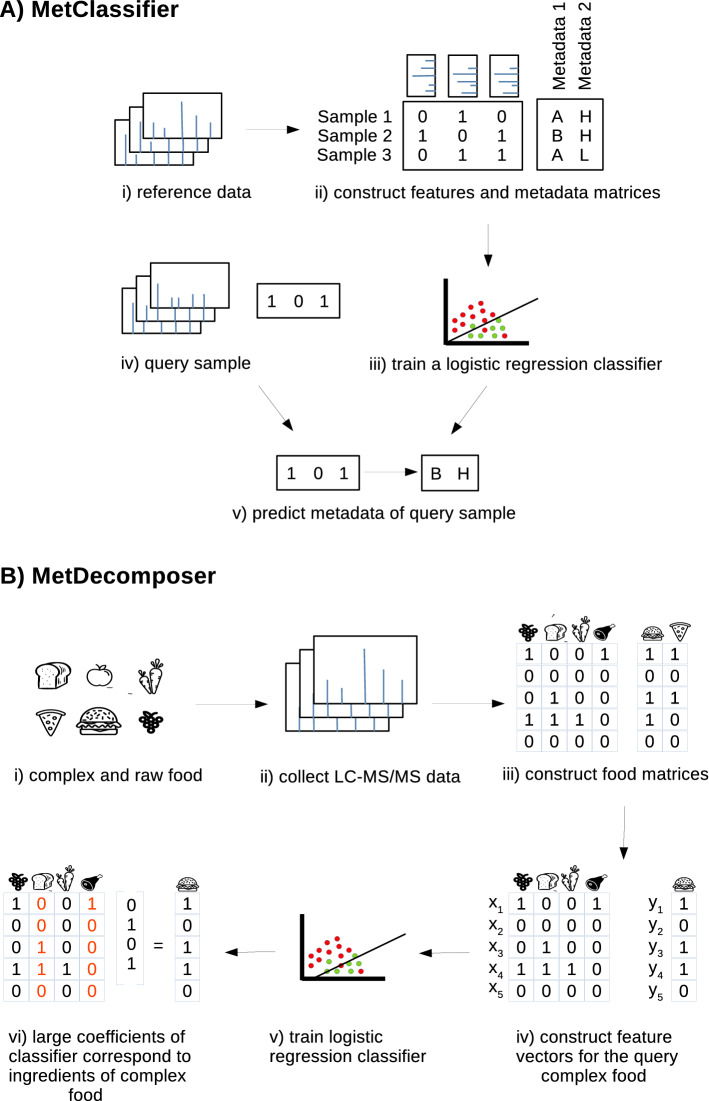
MetSummarizer pipeline. (**A**) MetClassifier, (i) starts with reference mass spectra of environmental/host-oriented samples. (ii) Mass spectrometry feature and metadata matrices are formed. (iii) A logistic regression classifier is trained for predicting phenotype from mass spectrometry features. (iv) Mass spectrometry feature vector is formed for query sample. (v) The classifier predicts phenotypes for query sample. (**B**) In MetDecomposer, starting from (i) complex and raw foods, (ii) LC-MS-MS data is collected. (iii) Matrices corresponding to the spectral features of raw and complex foods are formed. Then in order to find composition of a complex food, (iv) construct a feature vector for the complex food sample. (v) Train a logistic regression classifier to identify raw ingredients of the complex food. (vi) Large coefficients of the classifier correspond to ingredients of the complex food (i.e if the *i*th coefficient of classifier is large, then the *i*th ingredient in raw food matrix is present).

MetSummarizer has two components, MetClassifier and MetDecomposer. MetClassifier predicts the phenotype of samples in the following steps (Fig. 1A): (i) reference mass spectra of environmental/host-oriented samples are collected, (ii) mass spectrometry feature and phenotype metadata matrices are formed, (iii) logistic regression classifier is trained for predicting phenotype from mass spectrometry features, (iv) mass spectrometry feature vector is formed for query sample, and (v) the classifier predicts phenotypes for query sample. MetDecomposer, predicts the raw ingredient composition of complex foods in the following steps (Fig. 1B): starting from (i) complex and raw foods (ii) LC-MS-MS data is collected. Then (iii) matrices corresponding to the spectral features of raw and complex foods are formed. In order to find the ingredients of a complex food, (iv) construct a feature vector for the complex food sample and (v) train a logistic regression classifier to identify raw ingredients of the complex food. (vi) Large coefficients of the classifier correspond to ingredients of the complex food (i.e if the *ith* coefficient of classifier is large, then the *ith* ingredient in raw food matrix is present).

### Datasets

MetClassifier is trained on the dataset of 34,087 samples from ReDU. Each sample contains a binary vector encoding the absence/presence of 13,211 molecular features^5^ and is accompanied with annotations of 27 environmental, biological, and clinical phenotypes including taxonomy, biological sex, and disease status in the case of host oriented samples and lattitude, longitude, and depth/altitude in the case of environmental samples. REDU also reports the standards used in each of the datasets. MetDecomposer is tested on a dataset of 1852 raw and 1682 complex food samples coming from the Global FoodOmics Project (GFOP)^10,11^. We extracted 95,006 binary LC-MS-MS features from mass spectra of each food sample using MSCluster^12^. For each complex food sample, GFOP provides a list of raw ingredients.

### Increasing accuracy of prediction by aggregation of datasets

**Figure 2:**
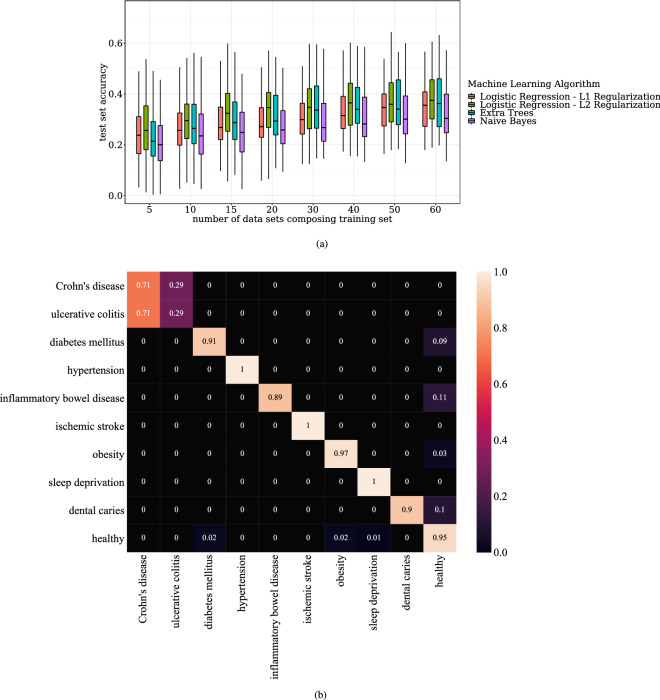
(**a**) Average test performance of MetClassifier as data is aggregated. Figure **a** shows the average test performance of several machine learning algorithms in prediction of life stage (early childhood, adolescence, early adulthood, middle adulthood etc.) as the number of datasets in the training set increases. The average is taken over twenty trials where in each trial the test set is composed of twenty randomly chosen datasetsand the training set is composed of the remaining datasets. Here if a particular dataset is used in the training/test set then all the samples associated with the dataset are used in the training/test set. (**b**) Accuracy of MetClassifier predictions for clinical phenotypes. Note the largest confusion is between Crohn’s disease andulcerative colitis, which are known to have similar symptoms.

Test accuracy of several machine learning algorithms for phenotype predictions increased as more samples and datasets were incorporated in the training data. Here, accuracy refers to the fraction of the test dataset classified correctly. Figure 2a illustrates average test accuracy of Extra Trees, Naive Bayes, Decision Trees, and Logistic Regression for prediction of life stage (early childhood, adolescence, early adulthood, middle adulthood etc.) versus the number of datasets used for training. Here if a particular dataset is used in training then all the samples associated with the dataset are used in training. Training on 10 datasets, the machine learning algorithms have on average less than 25% accuracy. Training on 60 datasets, the machine learning algorithms attain on average atleast 32.5% accuracy.

### Classification of clinical phenotypes

Figure 2b illustrates the accuracy of MetClassifier’s disease prediction from metabolomics data. Here we used 80% of human data from ReDU for training and 20% test. The data contains subjects with no disease (17206 subjects), Crohn’s disease (193 subjects), dental caries (24 subjects), diabetes mellitus (100 subjects), hypertension (20 subjects), inflammatory bowel disease (22 subjects), ischemic stroke (44 subjects), obesity (679 subjects), sleep deprivation (712 subjects), and ulcerative colitis (137 subjects). Supplementary Table S2 shows the predicted diseases and true diseases of samples from ReDU. Supplementary Table S3 shows the predicted life stage and true life stage of samples from ReDU.

### Batch effects

Datasets from the ReDU repository are acquired using various protocols from multiple laboratories. These protocols differ in standard molecules added to the samples (e.g. sulfadimethoxine versus none), extraction methods (e.g. methanol versus ethyl acetate), mass spectrometry instrument (e.g. Q Exactive versus Impact), etc. Using data collected by various protocols can lead to bias, especially in cases where some biological phenotypes are collected only using a single protocol. For example, MSV000083077 dataset contains data from obese subjects using sulfadimethoxine as a standard, while MSV000081832 contains data collected on healthy subjects without any standard. MetClassifier identified sulfadimethoxine, tris(2-butoxyethyl) phosphate, dehydroxynocardamine, and mucic acid as the top four biomarkers when classifying between MSV000083077 spectra and MSV000081832 spectra. In particular, sulfadimethoxine was identified as a biomarker that indicates samples are obese, which is an artifact of distinct data acquisition protocols. Such artifacts can not be detected unless an interpretable technique (e.g. logistic regression) is used. For example, when trained on 80% of MSV000083077 and MSV000081832 and tested on the other 20%, MetClassifier accuracy is 95%. However, when trained on the same datasets and tested on MSV000083462 (healthy data with sulfadimethoxine as internal standard), the accuracy drops to 20%. This is mainly because the classifier misclassifies all healthy data as obese due to the presence of sulfadimethoxine. There are several ways to avoid this bias. One way is to train the classifier on healthy/disease data from more diverse protocols. For example, if MSV000083462 were added to the training data, then the accuracy on test data increases to 94%. Another alternative is to encourage the community to standardize data acquisition protocols as much as possible. Finally, interpretable classification(i.e. logistic regression) allows for detection of the features that are significantly different between classes. MetClassifier reports these features and allows for discarding features that cause bias.

### Detecting raw ingredients of complex dishes

We applied MetDecomposer to decompose complex dishes from the Global FoodOmics database. Currently, this database contains well curated samples of 1852 raw ingredients and 1682 complex dishes. For each complex dish, MetDecomposer predicts the five most likely raw ingredients. Supplementary Table S1 shows a comparison of MetDecomposer predictions with the ingredients reported in the Global FoodOmics database on all complex dishes, and Table 1 shows results for a randomly selected subset of dishes. In order to prove the accuracy of our ingredient prediction algorithm, we have to show that the predicted ingredients of each mixture match up with the actual ingredients. We observed that on average, 1.57 out of top five predicted ingredients are correct or in the annotated ingredient list. In order to assess whether this overlap is statistically significant, we developed a random predictor that assigns random ingredients to each complex food. These predictions have an overlap of 0.05 out of top five predicted ingredients with actual ingredients, showing that the accuracy of MetSummarizer is statistically significant.

Part of the discrepancy between reports between MetDecomposer and the Global FoodOmics database could be explained by the fact that for some of the dishes, the ingredients reported in the Global FoodOmics database are incomplete or inaccurate. For example, in the case of “carrot in chicken biryani”, the reported ingredients are carrot, rice, chicken, and peas while MetDecomposer also predicts tomato, a likely ingredient of chicken biryani. Another source of discrepancy between the predicted and reported ingredients is due to the fact that the Global FoodOmics database currently does not include many of the raw ingredients due to the enormous diversity of dishes. In those cases, MetDecomposer usually predicts the raw ingredients available in the database that are most similar to the missing ingredients. For example, in the case of “gin”, the ingredients are gin, juniper berry, bulgarian rose and cucumber. None of these raw ingredients are available in Global FoodOmics database. In this case, MetDecomposer predicts buckwheat (a known ingredient of various alcohols) and acai berry, which are similar to the actual raw ingredients.

**Table 1:** Results of applying MetDecomposer to complex foods in Global FoodOmics database. For each dish, five top raw ingredients are predicted.

Dish	Dish ingredients	Predicted ingredients
Rice and chicken	rice, chicken	Rice, chicken, cake, cow liver, anchovies
Toddler’s solution	Nonfat milk, corn syrup, vegetable oil, sugar	Bread, peas, cow milk, whole milk, corn
Rice and chicken biryani	Rice, chicken	Pasta sauce, white rice, brown rice, basmati rice, boiled rice
Pizza	Tomato paste, grain, enriched flour, mozzarella cheese, pepperoni	Bread, chicken, cheese, mushroom, beef
Carrot in chicken biryani	Carrot, rice, chicken, peas	Chicken breaset, persian cucumber, peas, carrots, tomato
Strawberry greek yogurt	Pasteurized milk, cream, strawberry vanilla base	Cream cheese, cheese, sour cream, mushroom, milk
Prosciutto	Pig, salt, pepper, rice	Black pig, commercial pig, bovine, chicken, trout
Tomato sauce	Tomato, salt, basil, olive oil	Grashopper body, laurel, hemp, olive, tomato puree
Macaronni and cheese	Milk, cheese, macaronni	Wheat, cheddar cheese, mushroom, carrot
Strawberry cream cheese	Milk, cream, strawberry puree, whey protein,dried strawberry	Cream cheese, sour cream, strawberries, carrot, caviar
Lorimar	Sangiovese grape, merlot grape	Wine
Beet apple ginger juice	Beet, lemon, apple, ginger	Apple sauce, lemon peel, ginger, lime flesh
Strawberry jam	Strawberry, apple pectin, cane sugar, ascorbic acid	Granola bar, apple sauce, strawberry, banana
Primate mini biscuits	Soybean, corn, oats, beet, apple	Granola bar, apple sauce, soybean, grain mixture, edamame
Candied orange with chocolate	Candied orange, dark chocolate, cocoa butter, soy lecithin, vanilla	Milk chocolate nuts, orange juice, chocolate icing, soy milk, cheerios
Mazuri	Soybean, oat, beet, corn	Apple sauce, yeast, strawberries, soybeans, beef
Peanut butter	Milk chocolate, peanut butter, sugar, cornstarch, dextrose	Peanut, chocolate
Juice	Carrot, oranges, apple, lemon	Naval orange, mandarin, blueberry
Garlic knot	Dough, garlic, parmesan cheese, herbs	Bread roll, butter, cheese
Mashed potatoes	Potatoes, milk, butter, salt, pepper	Potato chip, potato puree, cheese pasta, filling of pistachio macaroon, egg
Outside of lemon macaroon	Sugar, almonds, egg white	Almonds, chicken soup, filling of pistachio macaroon
Pressed juice	Carrot, apple, spinach, romaine, parsley, ginger	Carrot juice, lemon peel, lemon flesh, grapefruit meat
Gin	Gin, juniper berry, bulgarian rose, cucumber	Buckwheat, goat milk, orange juice, cocoa powder, acai berry

## Discussion

Currently, fast and inexpensive diagnosis methods are not available for many diseases. Metabolomic data collected from various body-sites has the potential for revealing the molecular mechanism of disease, providing the path toward diagnosis. However, the majority of studies are limited to linking a single disease to its molecular biomarkers. MetSummarizer is the first method for systematic prediction of clinical/biological phenotypes by training on over thirty thousand metabolomics samples aggregated from over eighty studies. Currently MetSummarizer predicts disease with accuracy of eighty percent or higher. As the amount of annotated metabalomics data is expected to grow in the future, we expect this accuracy to improve. This belief is supported by an experiment in this paper showing machine learning accuracy of life stage prediction improved from 25 to 32.5% as the training data increased. As new datasets are added to ReDU, MetSummarizer will be periodically updated to increase the accuracy of predictions.

One of the main challenges of training on aggregate data is the bias introduced by using data acquired from different protocols. MetSummarizier alleviates this batch effect by using interpretable techniques capable of detecting biomarkers that support the classification, allowing for manual/automated exclusion of bias. MetSummarizer also features a technique for decomposing complex samples into their raw ingredients. Our results on the data from the Global FoodOmics project show that MetSummarizier correctly predicts 30% of the ingredients from complex dishes among its top five predictions. Currently MetSummarizer uses a rule based strategy for predicting the ingredients of complex foods. This rule based strategy is based upon the hypothesis that the molecular profile of complex food is nearly equal to the union of the molecular profile of its ingredients, and thus it could be sensitive to mis-annotations. Recently extreme multiclass classification techniques have enabled accurate label prediction for datasets with multiple labels. These methods could potentially enable more accurate prediction of ingredients even in the presence of mis-annotations.

For both the task of disease prediction and ingredient prediction, MetSummarizer only uses the presence/absence of small molecules. Our experiments show that MetSummarizer’s performance deteriorates if quantitative information is used. This might be due to the bias induced toward abundant features.

## Methods

### Overview of MetSummarizer

MetSummarizer consists of MetClassifier and MetDecomposer. MetClassifier predicts biological phenotypes of a sample given its metabalome. MetDecomposer detects the raw ingredients of complex samples.

### Retrieving features from LC-MS spectra

MetClassifier and MetDecomposer use features extracted from the raw spectra. In case of MetClassifier, the features are extracted by spectral library search of known molecules^2^, while in case of MetDecomposer the features are extracted using MSCluster^12^. Both spectral library search (based on cosine similarity) and MSCluster take advantage of intensities in fragment mass spectra. In both cases, the features are binary, where 1 represents the presence of a metabolomics feature, while 0 represents its absence.

### Training MetClassifier

Logistic regression model with $l_{1}$ norm regularization is trained on training data. $l_{1}$ norm regularization is used to enforce sparsity. This sparsity regularization prevents overfitting and allows the model to be interpretable. In regular logistic regression, the optimization criteria is

1 $$\begin{aligned} {{\min }} \ \ \ \sum _{t =1}^{T} L(f(x^{t}), y^{t}) \end{aligned}$$

where *t* indexes each training point, $y^{t}$ represents the true label of each training point, $x^{t}$ represents the features of each training point, *f* is a function that outputs a label given features $x^{t}$, and *L* refers to a loss function that is low when $f(x^{t})$ is equal to $y^{t}$ and high otherwise. Here we use $T=34{,}087$ training samples from ReDU. Currently, the default option for MetSummarizer is logistic regression based on 11 regularization. While l2 regularization outperforms 11 regularization on the task of predicting stage of life, 11 regularization leads to sparsity of logistic regression coefficients (only a few of the coefficients are non-zero) leading to a significantly more interpretable model, and facilitating detection of bias. The choice of method can be adjusted by the user.

Currently there is an imbalance between different classes for various phenotypes in ReDU. For example, among host oriented samples, over 90% belong to healthy individuals. Such an imbalance could result in misclassification of disease subjects to healthy. To avoid this we use a “balanced” approach^13^. Notice that each training point in (1) contributes the same amount to the total loss. We modify the objective function in (1) to the following:

2 $$\begin{aligned} {{\min }} \ \ \ \sum _{t =1}^{T} \frac{L(f(x^{t}), y^{t})}{b^{t}} \end{aligned}$$

where $b^{t}$ is the number of training points with label $y^{t}$. This way each disease or phenotype contributes the same amount to the objective function that we aim to minimize.

### Removing artifacts from data

In order to remove artifacts from the data, first an 11 logistic regression model is trained on the training data. Since the logistic regression coefficients are mostly zero due to 11 regularization, only a few features will have non-zero coefficients. If any of these features correspond to internal standards or artifacts relevant to experimental conditions, then they are removed and the model is retrained.

### Constructing raw ingredient and complex food matrices

First, raw and complex food matrices are formed. Each column of the raw matrix corresponds to a binary vector of a raw food, and each column of the complex matrix corresponds to the binary vector of a complex food. Each matrix has $T = 95{,}006$ rows. The raw and complex matrices have 1852 and 1682 columns, respectively.

### Finding ingredients of complex dishes

In order to find the ingredient composition of a complex food we make two modelling assumptions. We assume that (i) each complex food is composed of only a few ingredients, and (ii) the molecular profile of a complex food is nearly equal to the union of the molecular profile of its ingredients. Due to these two assumptions, we use an objective function exactly equivalent to that of logistic regression with 11 regularization:

3 $$\begin{aligned} \begin{aligned} \underset{\mathbf {x}}{{\min }}&\sum _{t=1}^{T} CrossEntropy\bigg (\mathbf{c}^{t}, Sigmoid\big ((\mathbf{D}{} \mathbf{x})^{t}\big )\bigg ) + \lambda |\mathbf{x}|_{1}\\ \end{aligned} \end{aligned}$$

where the minimization is over vector *x*, which approximates the abundance of raw ingredients in the complex dish. Here $\mathbf{D}$ is the raw ingredient matrix with 95,006 rows and 1852 columns, $\mathbf{c}$ is a binary vector of size 95,006 corresponding to the metabalome of a complex food. $\lambda$ is a positive scalar value and $|\mathbf{x}|_{1}$ denotes the sum of the absolute values of the entries in $\mathbf{x}$. Sigmoid is a monotonically increasing function that takes as input a scalar value and outputs a value between 0 and 1. Its functional form is the following:

4 $$\begin{aligned} Sigmoid(z) = \frac{1}{1 + \exp (-z)} \end{aligned}$$

CrossEntropy between a binary variable *y* and a real value $\hat{y}$ between 0 and 1 is defined as^14^:

5 $$\begin{aligned} CrossEntropy(y,\hat{y}) = -y\log (\hat{y}) -(1 -y)\log (1-\hat{y}) \end{aligned}$$

Increasing $\lambda$ forces the minimizer of (3) to satisfy the sparsity assumption. The CrossEntropy term ensures the union assumption holds. Due to the fact that the majority of entries in $\mathbf{c}$ are zero, optimizing (3) may lead to a solution that has low CrossEntropy whenever $\mathbf{c}_{t} =0$ but not when $\mathbf{c}_{t} =1$. This would result in the minimizer violating the union assumption. To avoid this, we use the “balanced” approach^13^ by defining the following optimization:

6 $$\begin{aligned} \begin{aligned} {{\min }}&\sum _{t=1}^{T} \frac{CrossEntropy\bigg (\mathbf{c}^{t}, Sigmoid\big ((\mathbf{D}{} \mathbf{x})^{t}\big )\bigg )}{b^{t}} + \lambda |\mathbf{x}|_{1}\\ \end{aligned} \end{aligned}$$

where $b^{t}$ is the number of entries in $\mathbf{c}$ with value $\mathbf{c}^{t}$. We solve (6) for increasing values of $\lambda$ until the minimizer of (6) has five non-zero entries. The algorithm then outputs the ingredients that correspond to these non-zero entries.

## Supplementary Information

Supplementary Information 1.

Supplementary Information 2.

Supplementary Information 3.

Supplementary Information 4.
