# Supplementary material for: Repository scale classification and decomposition of tandem mass spectral data
Source: Sci Rep. 2021 Apr 15;11:8314. doi: 10.1038/s41598-021-87796-6 (PMC8050247; doi:10.1038/s41598-021-87796-6)
Supplement: Supplementary file 1 — Supplementary Information 1. [file 41598_2021_87796_MOESM1_ESM.pdf]

# Repository Scale Classification and Decomposition of Tandem Mass Spectral Data

Mihir Mongia, Hosein Mohimani

Carnege Mellon Univerity
Computational Biology Department, School of Computer Science

## Supplementary Figures and Tables

**Classification of Biological Phenotype** We tested MetClassifier for simultaneous prediction of taxonomy, biological sex, life stage, and health status of host associated samples. Metclassifier achieved seventy eight percent accuracy. A random classifier achieves one percent accuracy due to the fact that there 85 combinations of taxonomy, biological sex, life stage, and health status (Figure 1).

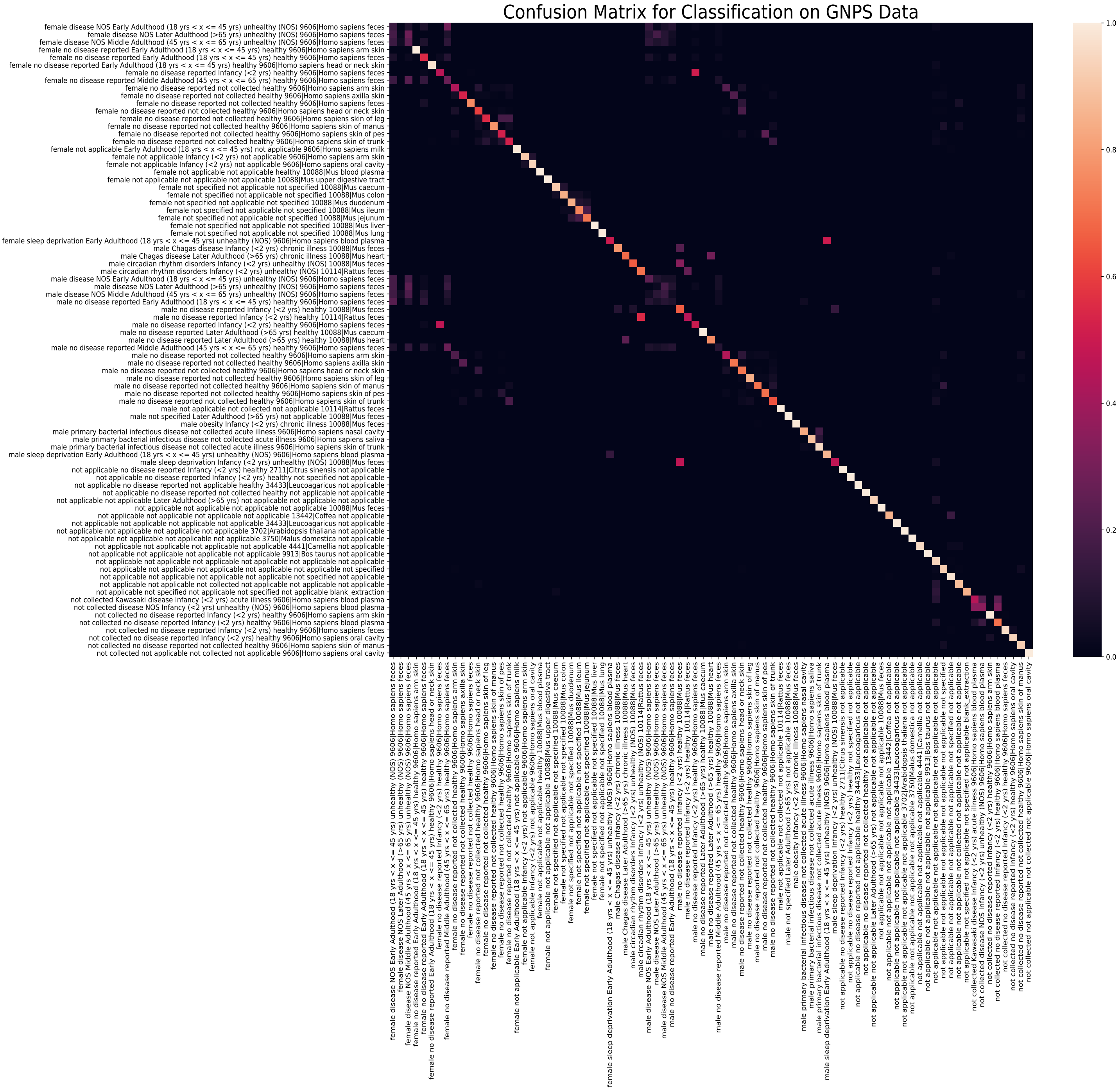


Figure 1: Confusion matrix of metclassifier for simultaenous prediction of taxonomy, biological sex, life stage and health status.

**Test accuracy for disease prediction of various algorithms** We compared the test performance of various algorithms on disease prediction. Logistic and extra trees regression achieve about 95% percent accuracy and perform better than Random Forest, Naive Bayes, and PLSD.

| Algorithm | Test Accuracy |
|---|---|
| l1 Logistic Regression | 94% |
| l2 Logistic Regression | 95% |
| Extra Trees | 95% |
| Random Forest | 88% |
| Naive Bayes | 91% |
| PLSD | 69% |

Table 1: Test accuracy of several standard machine learning algorithms on clinical phenotype.

**Confusion matrix of disease prediction with l2 logistic regression** Accuracy of l2 logistic regression predictions for clinical phenotypes. Note the largest confusion is between Crohn's disease andulcerative colitis, which are known to have similar symptoms. The confusion matrix is largely similar to that of l1 logistic regression (shown in main text)

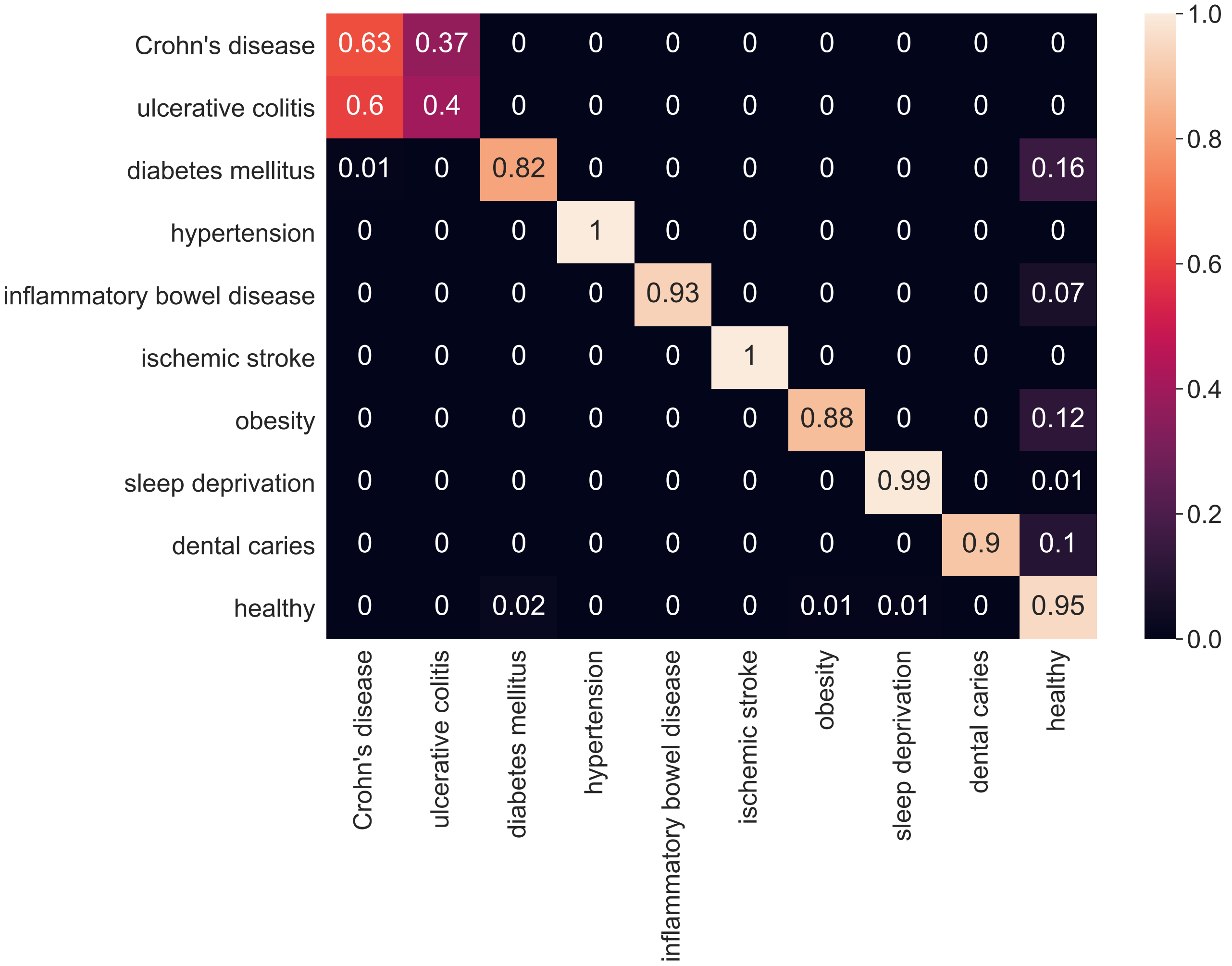


Figure 2: Accuracy of l2 logistic regression predictions for clinical phenotypes. Note the largest confusion is between Crohn's disease andulcerative colitis, which are known to have similar symptoms. The confusion matrix is largely similar to that of l1 logistic regression (shown in main text).

## Supplementary Tables attached as Excel sheets

Supplementary Table S1 shows a comparison of MetDecomposer predictions with the ingredients reported in the Global FoodOmics database on all complex dishes.

Supplementary Table S2 shows the predicted diseases and true diseases of samples from ReDU.

Supplementary Table S3 shows the predicted life stage and true life stage of samples from ReDU.
